# Supplementary material for: Western tropical Pacific multidecadal variability forced by the Atlantic multidecadal oscillation
Source: Nat Commun. 2017 Jul 7;8:15998. doi: 10.1038/ncomms15998 (PMC5504304; doi:10.1038/ncomms15998)
Supplement: Supplementary Information [file ncomms15998-s1.pdf]

Title of file for HTML: Supplementary Information
Description: Supplementary Figures

Title of file for HTML: Peer Review File
Description:

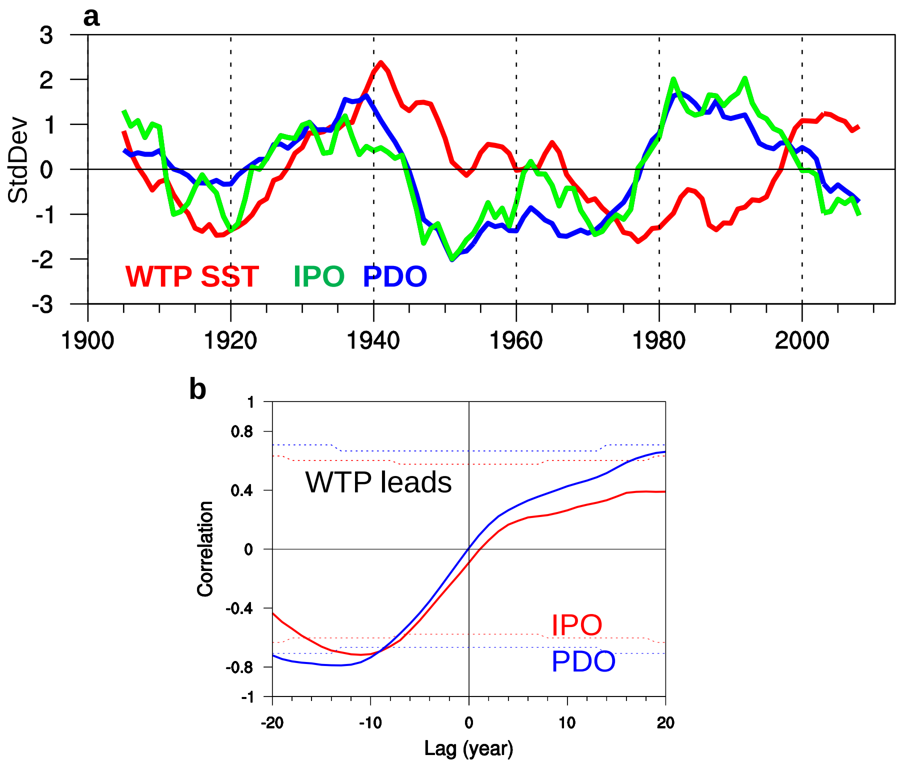

Supplementary Figure 1 IPO (or PDO) and the WTP SST decadal variability. (a) 11-yr running mean time series of IPO, PDO and detrended WTP SST indices for 1900–2013. All smoothed indices are scaled to unit variance. The PDO index was obtained from the University of Washington (http://jisao.washington.edu/pdo/PDO.latest). The IPO index is the tripole IPO index developed by Henley et al. (2015), available at http://www.esrl.noaa.gov/psd/data/timeseries/IPOTPI/. The correlations of the IPO and PDO with the decadal WTP are –0.09 and 0.01, respectively. (b) Cross correlations as a function of the time lag (year) of the low-pass filtered WTP SST with the IPO (red) and PDO (blue) indices. Positive (negative) lags indicate that the IPO/PDO (WTP SST) is leading. The dashed lines are the 95% confidence levels based on the effective numbers of degrees of freedom.

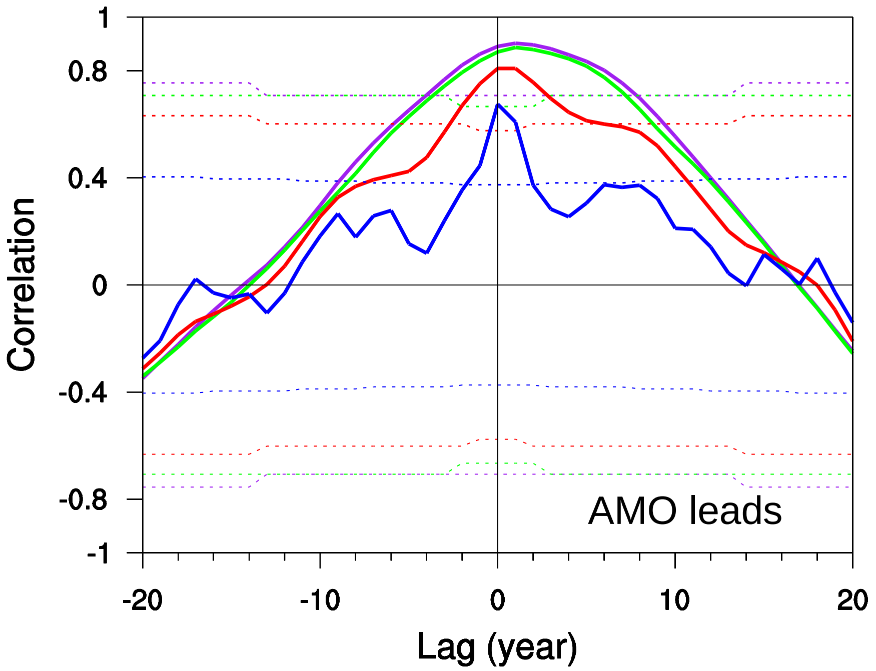

Supplementary Figure 2 Lead–lag correlation between the WTP SST and AMO indices (shown in Fig. 1b) for different smoothing time scales. The blue, red, green and pink lines are for unsmoothed, 5-yr, 9-yr and 11-yr running mean time series, respectively. The definitions of the WTP SST and AMO indices are given in the text. Positive (negative) lags indicate that the AMO (WTP SST) is leading. The dashed lines are the 95% confidence levels based on the effective numbers of degrees of freedom.

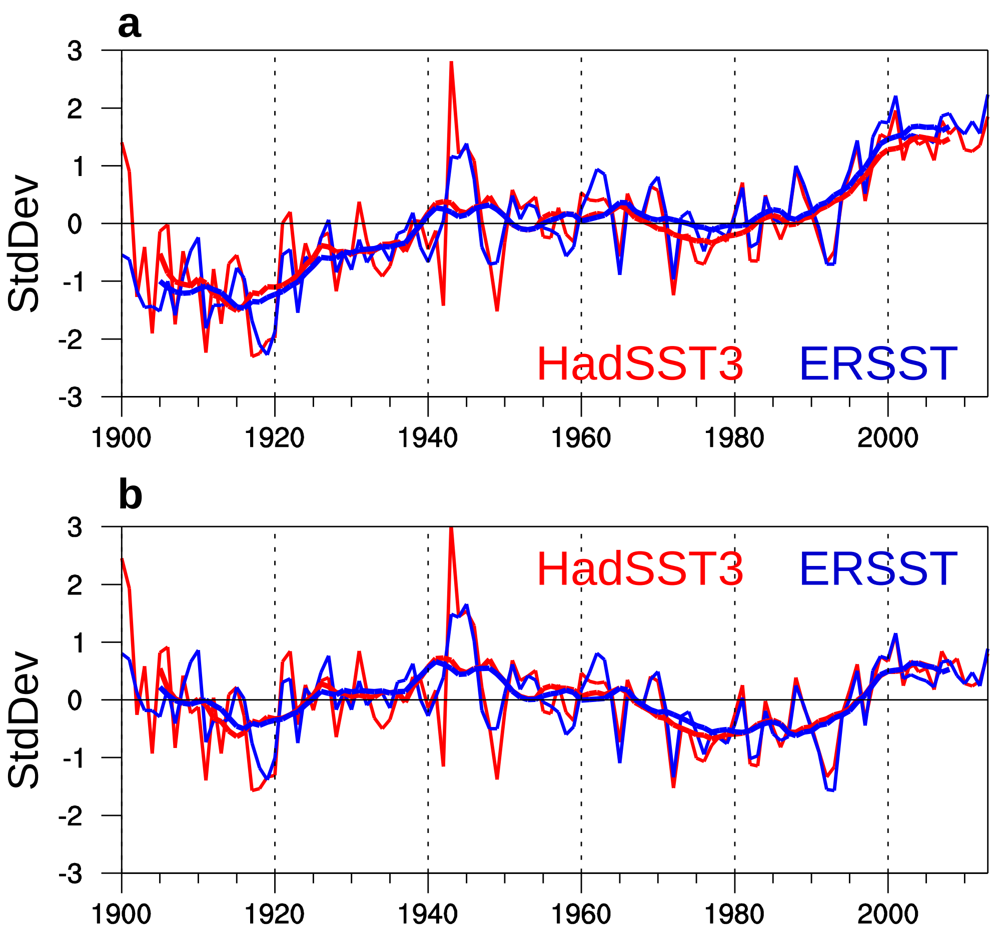

Supplementary Figure 3 WTP SST index from different SST data sets. (a) Normalized time series of the WTP SST index from the HadSST3 and ERSST data sets (thin lines) and the 11-yr running averages (thick lines) for the period 1900–2013. (b) As in (a), but for the data with removal of the long-term trends.

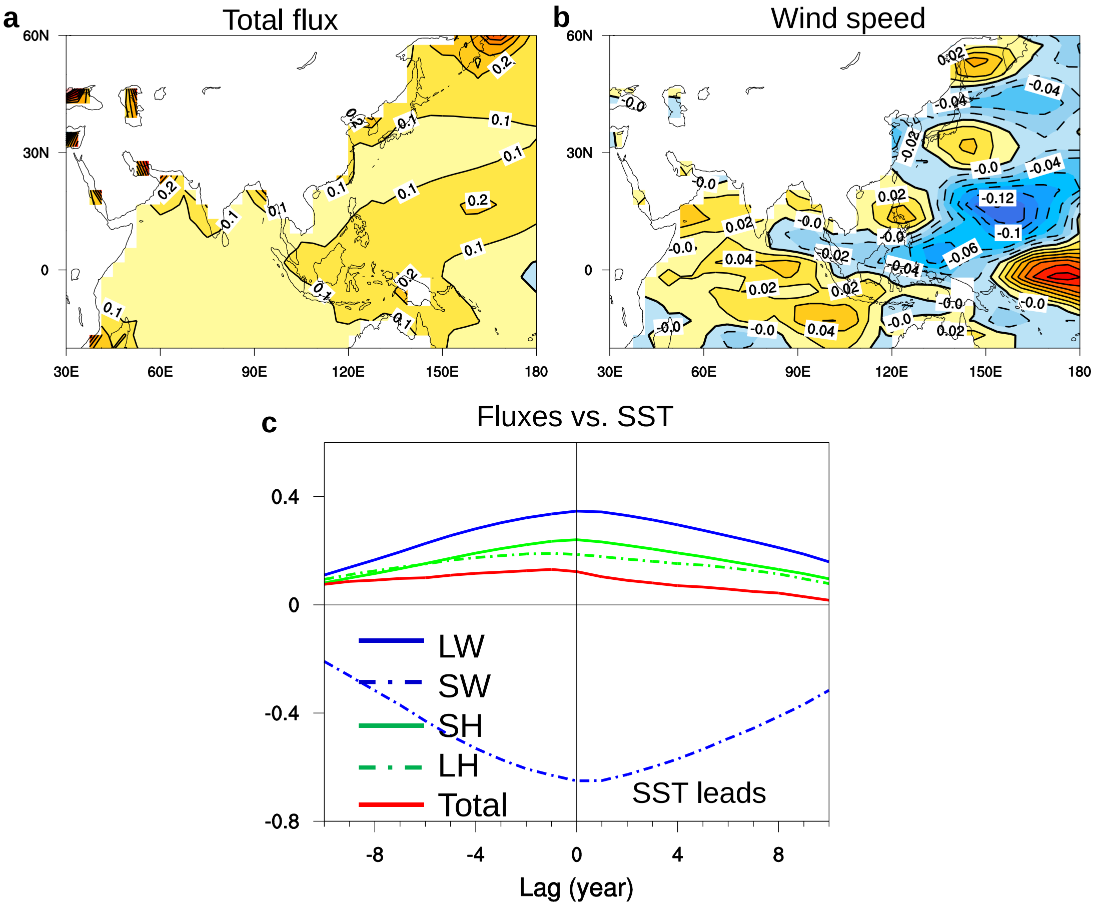

Supplementary Figure 4 Surface heat fluxes and wind speed associated with the WTP decadal variability in the ATL_VARMIX simulations. (a) Regressions of total net surface heat flux (units: W m^{-2}) on the normalized WTP SST index at decadal time scales. (b) As in (a) but for the surface wind speed (units: m s^{-1}). (c) Lagged regressions of the WTP regional averages of surface heat fluxes (units: W m^{-2}) onto the normalized WTP SST index at decadal time scales. Positive (negative) lags indicate that SST (fluxes) is leading. All fluxes are defined to be positive downward. The long-term linear trends for 1900–2013 in all variables were removed before the regression analysis.

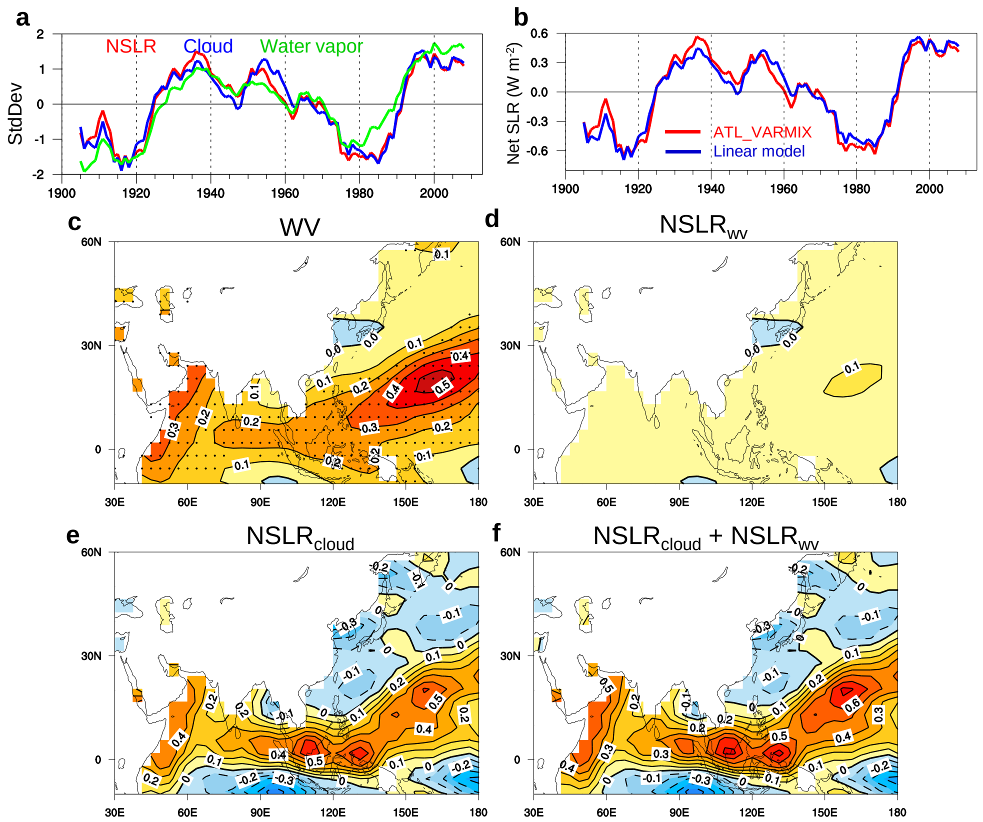

Supplementary Figure 5 Effects of cloud feedback and water vapor (WV) feedback on the decadal net surface longwave radiation (SLR) anomalies in the ATL_VARMIX simulations. (a) Time series of the simulated decadal net SLR, convective cloud cover and column-integrated WV anomalies averaged over the WTP region. The time series are filtered using an 11-year running mean, and all smoothed indices are scaled to unit variance. (b) The simulated decadal net SLR anomalies (units: W m^{-2}) averaged over the WTP region and the linear model fit of the SLR based on the decadal cloud cover and column-integrated WV anomalies. The linear model is developed as: SLR = $a\times$ Cloud + $b\times$WV, where the coefficients a = 0.77 W m^{-2}/% and b = 0.21 W m^{-2}/(kg m^{-2}) are determined empirically by multiple linear regression based on the simulation data over the period of 1900–2013, so that the regression error of the linear model is minimized. (c) Regression map of column-integrated WV (units: kg m^{-2}) on the normalized WTP SST index at decadal time scales. Dots indicate the regressions significant at the 95 % confidence level. (d) Contribution from WV feedback to the decadal SLR anomalies (units: W m^{-2}) in association with the WTP multidecadal variability, as estimated by using the linear model (multiplying the column-integrated WV anomalies in (c) by the coefficient b). (e) As in (d), but for the contribution from cloud feedback, as estimated by multiplying the convective cloud cover anomalies in Fig. 4f by the coefficient a. (f) The total contribution from cloud feedback and WV feedback to the decadal SLR anomalies obtained by adding (d) to (e).

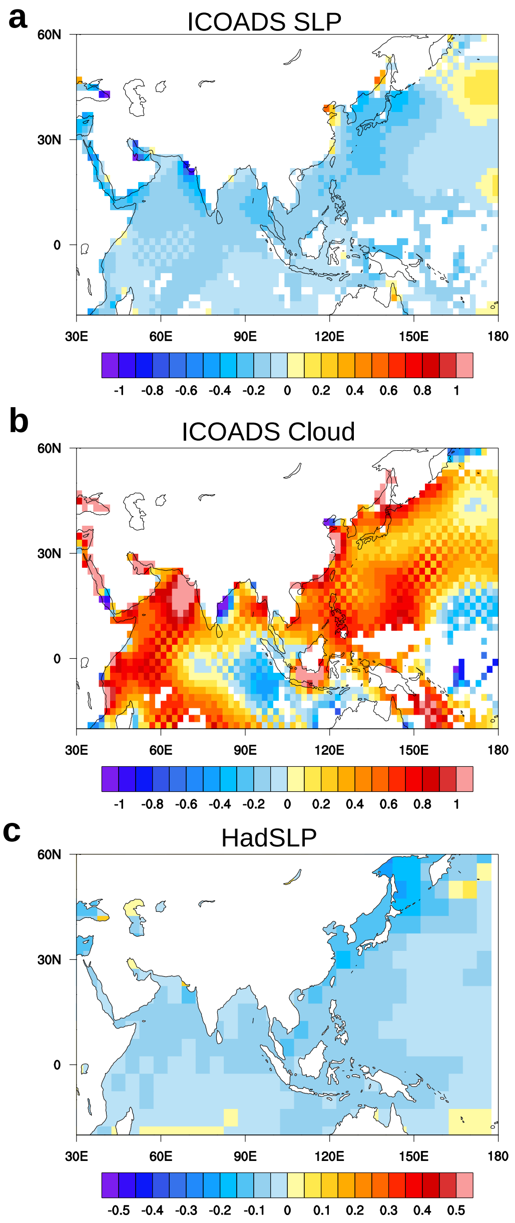

Supplementary Figure 6 Observational evidence for the SST–SLP–cloud–longwave radiation positive feedback over the WTP region as revealed by SLP and cloud cover fields. (a) Regressions of ICOADS SLP (units: hPa) on the WTP SST index at decadal time scales. (b) As in (a) but for the ICOADS cloud cover (units: %). (c) As in (a), but for the SLP data derived from HadSLP dataset. The long-term linear trends for 1900–2013 in all variables were removed before the regression analysis.

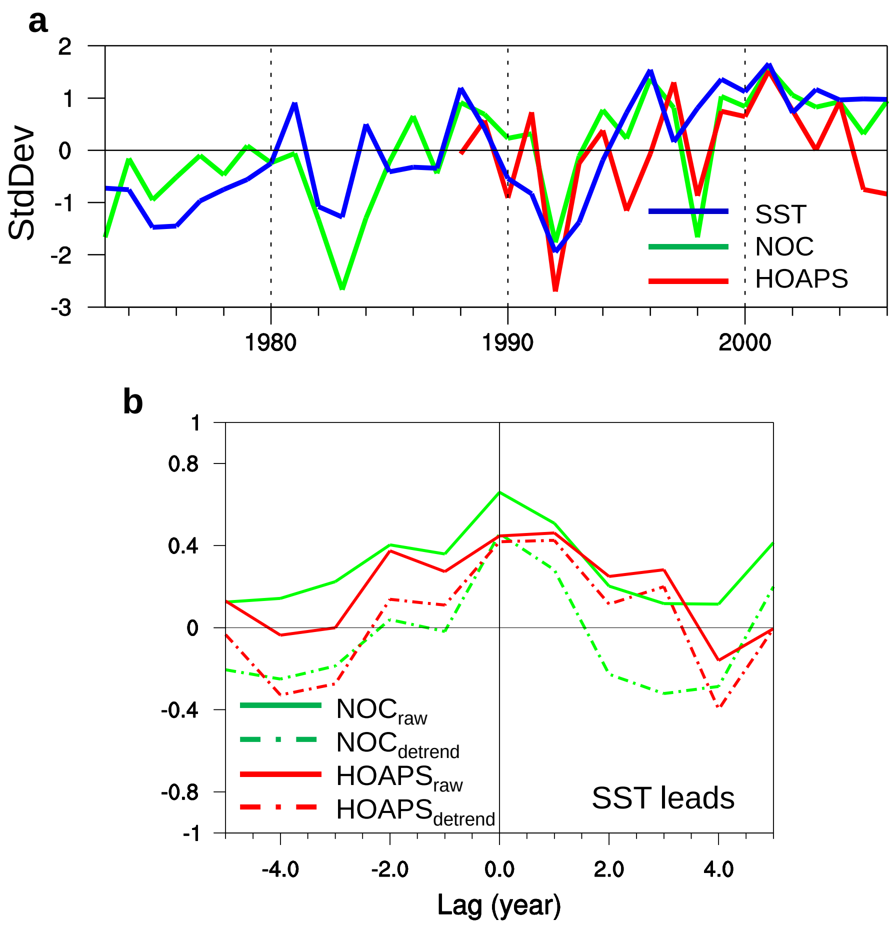

Supplementary Figure 7 Observational evidence for the SST–SLP–cloud–longwave radiation positive feedback over the WTP region as revealed by the longwave radiation field. (a) Time series of the WTP SST index (blue) and the WTP regional averages of net surface longwave radiation anomalies from HOAPS (red) and NOC (green) datasets. Net surface longwave radiation is defined positive downward. All time series are normalized to unit variance. (b) Lagged correlations of the WTP regional averages of net surface longwave radiation anomalies with the WTP SST index. Positive (negative) lags indicate that the SST (radiation) is leading.

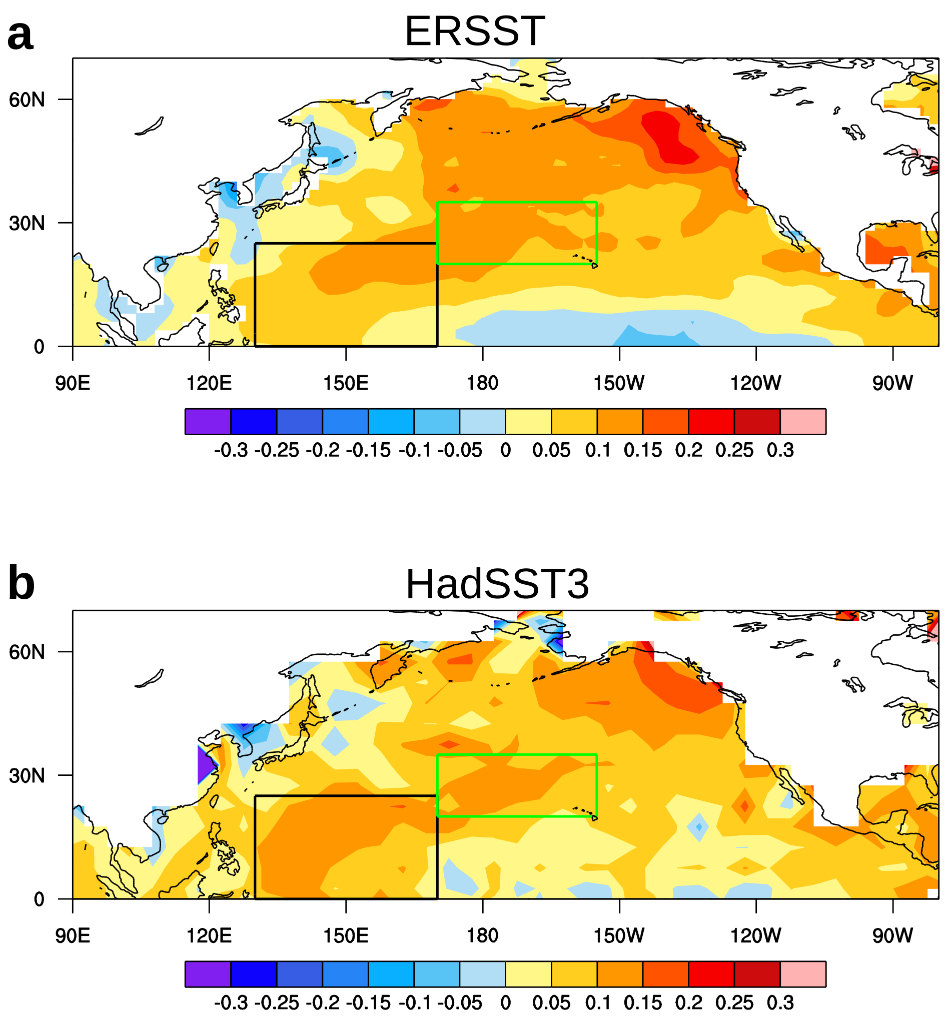

Supplementary Figure 8 The Pacific SST anomalies associated with the AMO. Regressions of Pacific SST north of equator (units: K) on the normalized AMO index for 1900–2013 at decadal time scales in the (a) ERSST and (b) HadSST3 datasets. The long-term linear trends in SST data were removed prior to the regression analysis. The black box indicates the region used to define the WTP (0°–25°N, 130°–170°E) while the green box is used to define the SNP region (20°N–35°N, 170°E–155°W).

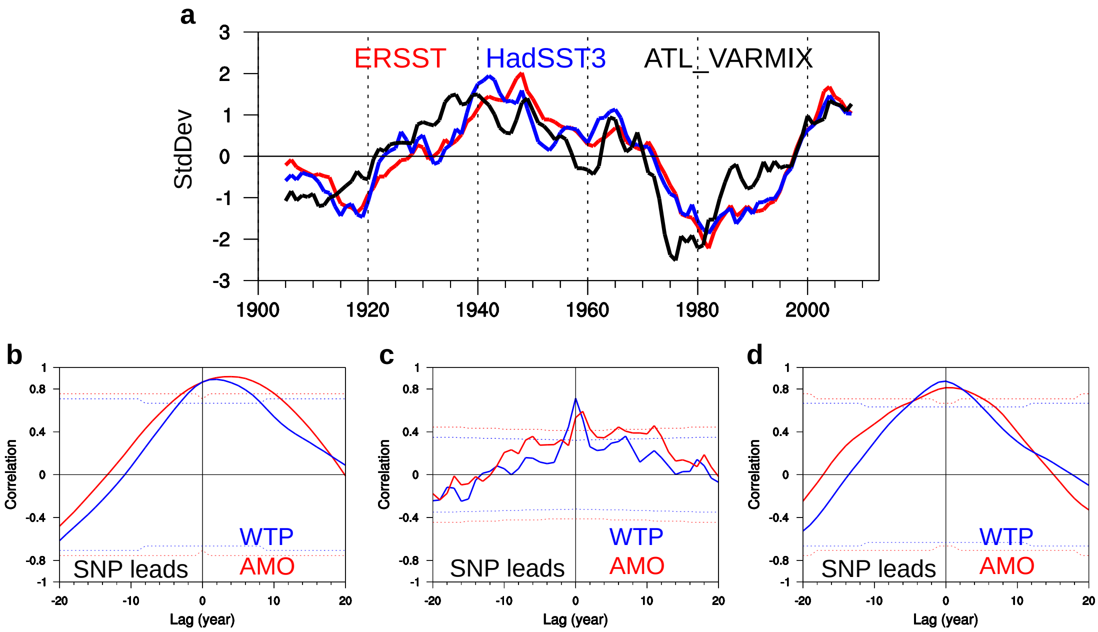

Supplementary Figure 9 Phase relationship of the SNP decadal SST anomalies with the WTP multidecadal variability and AMO in observations and ATL_VARMIX simulation. (a) Time series of the decadal SST anomalies averaged over the SNP region (20°N–35°N, 170°E–155°W) in the ERSST, HadSST3 and ATL_VARMIX datasets. The time series of SST anomalies are linearly detrended, filtered using an 11-year running mean, and normalized to unit variance. (b) Cross correlations as a function of the time lag (year) of the SNP decadal SST anomalies in ERSST with the AMO (red) and WTP SST multidecadal variability (blue) for the period 1900–2013. Positive (negative) lags indicate that the WTP SST/AMO (SNP SST) is leading. The dashed lines are the 95% confidence levels based on the effective numbers of degrees of freedom. (c) As in (b), but for the observed unfiltered time series of AMO, SNP and WTP SST anomalies, to further support the simultaneous relationship of the SNP SST with the AMO and WTP SST. (d) As in (b), but for the results in the ATL_VARMIX simulation.

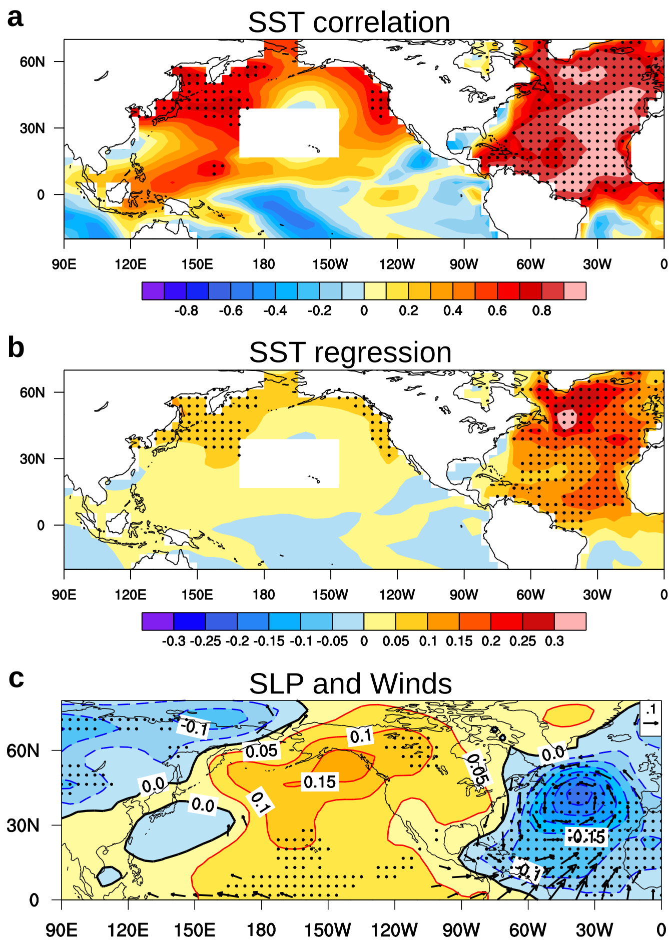

Supplementary Figure 10 Simulated Pacific response to the AMO in ATL_VARMIX_SNPCLIM simulation (see Methods for experiment description). (a) Correlation map between Pacific–Atlantic SST and the AMO at decadal time scales. Dots indicate the correlations significant at the 95 % confidence level. (b) Regression map of Pacific–Atlantic SST (units: K) on the normalized AMO index at decadal time scales. (c) Regressions of SLP (shading, units: hPa) and surface winds (vectors, units: m s^{-1}; omitted below 0.1 m s^{-1}) on the normalized AMO index at decadal time scales. In (b) and (c), dots indicate the regressions significant at the 95 % confidence level. The long-term trends in all variables were removed prior to the correlation and regression analyses.
